# Retraction Note: LncRNA LBX2-AS1 promotes colorectal cancer progression and 5-fluorouracil resistance

**DOI:** 10.1186/s12935-023-02881-2

**Published:** 2023-02-23

**Authors:** Yu-Nan Ma, Yong-Gang Hong, Guan-Yu Yu, Si-yuan Jiang, Bo-lun Zhao, An Guo, Yao Wang, Xiao-ming Cui, Li-Qiang Hao, Hao Zheng

**Affiliations:** 1 Key Laboratory of Carcinogenesis and Translational Research (Ministry of Education), Department of Laboratory Animal, Peking University Cancer Hospital & Institute, Beijing, 100142 China; 2 Department of Colorectal Surgery, Changhai Hospital, Second Military Medical University, Shanghai, 200433 China; 3 School of Nursing, Second Military Medical University, Shanghai, 200438 China; 4 School of Nursing, Dalian University, DalianLiaoning, 116000 China; 5 Department of Reproductive Heredity Center, Changhai Hospital, Second Military Medical University, Shanghai, 200433 China; 6 Shanghai Key Laboratory of Hepatobiliary Tumor Biology (EHBH), Shanghai, 200438 China; 7 State Key Laboratory of Pathogen and Biosecurity, Beijing Institute of Microbiology and Epidemiology, Beijing, People’s Republic of China; 8 Third Department of Hepatic Surgery, Eastern Hepatobiliary Surgery Hospital, Second Military Medical University, Shanghai, 200438 China

**Retraction Note: Cancer Cell Int (2021) 21:501** **https://doi.org/10.1186/s12935-021-02209-y**

The Editors-in-Chief have retracted this article because the cell lines used in this study (HCT116 and SW480) were found by the authors to be contaminated with HeLa cells. As a result, the Editors-in-Chief no longer have confidence in the data.

The authors Guan-Yu Yu and Yao Wang have not responded to any correspondence from the editor/publisher about this retraction. All other authors agree to this retraction.

